# Inhibition of Ceramide *De Novo* Synthesis with Myriocin Affects Lipid Metabolism in the Liver of Rats with Streptozotocin-Induced Type 1 Diabetes

**DOI:** 10.1155/2014/980815

**Published:** 2014-02-19

**Authors:** Krzysztof Kurek, Patrycja Wiesiołek-Kurek, Dominika M. Piotrowska, Bartłomiej Łukaszuk, Adrian Chabowski, Małgorzata Żendzian-Piotrowska

**Affiliations:** ^1^Department of Physiology, Medical University of Bialystok, Ulica Mickiewicza 2C, 15-222 Białystok, Poland; ^2^Department of Public Health, Medical University of Bialystok, Poland

## Abstract

Nowadays diabetes is one of the most common metabolic diseases. Sphingolipids, which are vitally important constituents of intracellular signal transduction pathways, may be among the most pathogenic lipid moieties intermingled in the origin and development of diabetes. It is now well established that inhibition of *de novo* ceramide synthesis with myriocin exerts positive effects on lipid metabolism and glucose homeostasis in type 2 diabetes mellitus animal models. However, its influence on type I diabetes still remains unknown. Therefore, the scope of this paper is to fulfill that particular gap in our knowledge.

## 1. Introduction

Diabetes is presently one of the most common metabolic diseases with approximately 300 million people affected globally in the year 2011. Furthermore, its incidence is increasing rapidly, and according to the World Health Organization (WHO) even 400 million people will suffer from diabetes mellitus by 2030 [1]. Thus, the search for new methods of diabetes treatment is a pressing issue.

The group of bioactive molecules, sphingolipids, are involved in numerous cellular processes, ranging from proliferation and differentiation of the cells to inflammatory responses and cellular apoptosis. Ceramide is a central molecule of sphingolipid metabolism. It is a sphingosine-based lipid moiety, which acts as a second messenger in a sphingomyelin (SM) signal transduction pathway through the activation of many kinases, phosphatases, and transcription factors [2]. Ceramide can be generated as a result of plasma membrane sphingomyelin hydrolysis, through activation of neutral or acidic isoform of enzymes sphingomyelinases (nSMase and aSMase), or it is synthesized within the endoplasmic reticulum in the so-called *de novo* pathway. The first step of this latter pathway, catalyzed by the enzyme serine palmitoyltransferase (SPT), encompasses condensation of amino acid serine and palmitoyl-CoA and formation of 3-ketosphinganine, which is then rapidly reduced to sphinganine (SFA). Afterwards, sphinganine is acylated to form dihydroceramide, which is converted into ceramide by the addition of trans-4,5-double bond. Ceramide can be converted into other sphingolipids by its degradation processes catalyzed by enzymes ceramidases. Three main isoforms of these enzymes: neutral, alkaline, and acidic have been previously described. Also ceramide derivatives, bioactive lipids, such as sphingosine (SFO) and sphingosine-1-phosphate (S1P), can influence cellular growth, differentiation, and programmed cell death and, additionally to ceramide, they also may be involved in the pathogenesis of type 1 and type 2 diabetes mellitus [3, 4].

Intracellular ceramide accumulation predisposes individuals to development of diabetes by impairment of insulin responsiveness. Studies by Holland et al. [5] revealed that when added to cell cultures (hepatocytes, myotubes, or adipocytes) ceramide analogs inhibit glucose uptake and glycogen synthesis [5]. The mechanism underlying these effects is based on inhibition of Akt/protein kinase B (Akt/PKB) action by ceramide moieties. First of all, ceramide blocks the translocation of the Akt/PKB from cytosol to plasma membrane [6]. Moreover, it promotes dephosphorylation and inactivation of Akt/PKB by protein phosphatase 2A [7, 8]. Finally, ceramide acutely inhibits GLUT4 translocation to plasma membrane [9]. Those previously described mechanisms lead to the onset and progression of insulin resistance and subsequent type 2 diabetes. Conversely, in cases of type 1 diabetes ceramide is responsible for pancreatic *β*-cells dysfunction. Numerous studies have proved that produced in response to inflammatory cytokines (such as tumor necrosis factor *α* (TNF*α*) or interleukin 1 (IL1)) ceramide inhibits insulin gene expression, blocks *β*-cells proliferation, and induces *β*-cells apoptosis [10–12]. On the other hand, ceramide derivative S1P (sphingosine-1-phospate) stimulates *β*-cells growth and promotes insulin secretion [13, 14]. Summarizing presented literature sources, ceramide should be considered as an important pathogenic factor involved in the development of type 1 diabetes.

Plasma free fatty acids (FFAs) play an important role in the induction of insulin resistance in the liver and skeletal muscle tissue and many studies confirmed interaction between FFAs and insulin signaling pathway [15]. Among FFAs, palmitic acid is considered as one of the major factors in the induction of insulin resistance [16]. Interestingly, palmitate is also a main substrate for ceramide *de novo *synthesis. FFAs (by activating *de novo* pathway) and TNF*α* (by activating nSMase and aSMase and *via* activation of *de novo* pathway) stimulate the accumulation of ceramide and its metabolites in different tissue types of diabetic rodents and humans [9]. Another important factor responsible for the pathogenesis of diabetes is intracellular accumulation of diacylglycerols (DAG) and triacylglycerols (TG) moieties. The accretion of hepatic DAG content could be a result of prolonged, intensified FFAs release from insulin resistant white adipose tissue [17]. The latter, in tandem with decreased mitochondrial *β*-oxidation rate and increased hepatic *de novo* lipogenesis, is likely to be the factor contributing to overaccumulation of DAG in hepatocytes [18].

*Isaria sinclairii, Myriococcum albomyces*, and *Mycelia sterilia* are fungi traditionally used in Chinese medicine to achieve eternal youth [19]. A drug called myriocin, isolated from these fungi, is a potent and highly selective SPT inhibitor [20]. Previously published data have showed that SPT inhibitor myriocin may be of potential use for treatment of selected cardiovascular diseases, such as atherosclerosis [21, 22]. Other studies provided evidence that inhibition of ceramide *de novo* synthesis with myriocin improved glucose homeostasis and enhanced whole-body insulin responsiveness in rodent models of type 2 diabetes [23]. Moreover, in a recently published study we have demonstrated that myriocin reduced body weight, ameliorated glucose homeostasis, and reversed hepatic steatosis in diet induced NAFLD (nonalcoholic fatty liver disease) [24]. However, to the best of our knowledge, effect of myriocin on lipid metabolism in case of severe hyperglycemia induced by streptozotocin (chemical compound toxic to the insulin-producing pancreatic *β*-cells) remains unexplained. Thus, in this study we evaluated the effect of SPT inhibitor myriocin on glucose homeostasis and lipid metabolism in the liver of rats with streptozotocin-induced type 1 diabetes. In addition, since diabetes is known to be accompanied with elevation of plasma free fatty acids concentration, we also examined whether the inhibition of ceramide *de novo* synthesis affects plasma FFAs level.

## 2. Materials and Methods

### 2.1. Animal Model

The experiment was carried out on male Wistar rats (*n* = 8 per group) fed *ad libitum* on standard rodent diet and bred in approved animal holding facilities (at 21-22°C, at stable humidity, on a reverse 12 h/12 h light-dark cycle, with unrestricted access to water and food). The animals were randomly divided into four groups:

1. “control” (C),
2. “myriocin” (M),
3. “diabetic” (D),
4. “diabetic + myriocin” (D + M).

Animals assigned to “M” group were treated with myriocin (given by intraperitoneal injections of 0.3 mg/kg of body weight) for 7 days. Rats assigned to the “D” group were given intravenously streptozotocin (Sigma) dissolved in citric buffer (pH 4.5) at a dose of 80 mg·kg^−1^. Finally, rats in “D + M” group were given streptozotocin and myriocin (for 7 days after first week since diabetes onset). After an overnight fasting rats were anaesthetized by intraperitoneal injection of pentobarbital (80 mg/kg of body weight) and sacrificed. Samples of the liver were excised, immediately freeze-clamped with aluminum tongs, and stored at a −80°C temperature until further analyses. Samples of blood from abdominal aorta were taken to measure levels of glucose, insulin, and FFAs. Animal maintenance and treatment were approved by the Ethical Committee for Animal Experiments at the Medical University of Bialystok.

### 2.2. Plasma Glucose, Insulin, and Free Fatty Acids Concentrations

Fasting serum glucose level was measured with Accu-chek (Bayer, Germany) glucose meter. Fasting serum insulin level was evaluated with chemiluminescence with commercial available ELISA kit (Abbot, USA). Plasma free fatty acids concentration was determined using the method described by Bligh and Dyer [25].

### 2.3. Sphingomyelin Content

The liver samples were pulverized in an aluminum mortar precooled previously in liquid nitrogen. The powder was then transferred to a tube containing methanol and 0.01% butylated hydroxytoluene (Sigma) as an antioxidant. Lipids were extracted by the method described by Bligh and Dyer [25]. Sphingomyelin was then isolated by means of thin-layer chromatography (TLC). Briefly, gel bands, corresponding to the standard, were scrapped off the plates and transferred into screw-cap tubes which contained pentadecanoic acid (Sigma-Aldrich, USA) as an internal standard. Sphingomyelin fatty acids were then transmethylated and subsequently analyzed by means of gas-liquid chromatography. A Hewlett-Packard 5890 Series II system, equipped with a double flame ionization detector and Agilent CP-Sil 88 capillary column (100 m, internal diameter of 0.25 mm), was used. The content of sphingomyelin is presented as the sum of individual fatty acid species of the assessed fraction and it was expressed in nanomoles per gram of the tissue.

### 2.4. Ceramide Content

A small (50 *μ*L) volume of the chloroform phase, containing lipids extract, was transferred to a fresh tube which contained internal standard (C17-sphingosine (Avanti Polar Lipids, UK)). Ceramide present in the organic phase was hydrolyzed in 1 M KOH in 90% methanol at 90°C for 60 min. The content of free sphingosine, liberated from ceramide, was next analyzed by means of HPLC. The calibration curve was prepared using N-palmitoylsphingosine (Avanti Polar Lipids, UK) as a standard. The chloroform extract used for the analysis of ceramide level also contains small amounts of free sphingoid bases. Therefore, the content of ceramide was corrected for the level of free sphingosine determined in the same sample.

### 2.5. Sphingosine, Sphinganine, and Sphingosine-1-phosphate Content

The ceramide derivatives were measured according to the method described by Min et al. [26]. Prior to samples homogenization and ultrasonication, internal standards (C17-sphingosine and C17-S1P (Avanti Polar Lipids, USA)) were added. The sphingoid bases were converted to their o-phthalaldehyde derivatives and analyzed on a HPLC system (ProStar, Varian, Inc., USA) equipped with a fluorescence detector and C18 reversed-phase column (Varian, Inc., OmniSpher 5, 4.6 mm × 150 mm).

### 2.6. Neutral and Acidic Sphingomyelinase Activity

Protein content was measured in all homogenates prior to enzymatic analysis with the BSA protein assay kit (Sigma-Aldrich, USA). As a standard, bovine serum albumin (fatty acid free (Sigma-Aldrich, USA)) was used.

The activity of neutral and acid isoforms of sphingomyelinase was determined according to Liu and Hannun [27], with the use of radiolabeled substrate [N-methyl-14C]-SM (Perkin-Elmer Life Sciences, USA). The product of reaction, 14C-choline phosphate, was extracted with CHCl3/methanol (2, 1; v/v), transferred to scintillation vials, and counted using a Packard TRI-CARB 1900 TR scintillation counter.

### 2.7. Alkaline, Neutral, and Acidic Ceramidase Activity

The activities of neutral and alkaline ceramidases were measured by the method described by Nikolova-Karakashian and Merrill Jr. [28]. The activity was determined with the use of radiolabeled [N-palmitoyl-1-14C]-sphingosine (Moravek Biochemicals, USA) as a substrate. Unreacted ceramide and liberated 1-14C-palmitate were separated with the basic Dole solution (isopropanol/heptane/1 N NaOH, 40, 10, 1; v/v/v). Radioactivity of the 1-14C-palmitate was measured by scintillation counting.

### 2.8. DAG and TG Content

The liver samples were pulverized in an aluminum mortar precooled in liquid nitrogen. The powder was transferred to a glass tube and lipids were extracted using the Bligh and Dyer method [25]. The fractions of total triacylglycerols and diacylglycerols were separated by thin-layer chromatography (TLC). Individual fatty acid methyl esters were identified and quantified according to the retention times of standard by gas liquid chromatography (Hewlett-Packard 5890 Series II gas chromatograph, with a Varian CP-SIL capillary column (50 m × 0.25 mm internal diameter) and flame ionization detector (FID) (Agilent Technologies, USA). Total diacylglycerol and triacylglycerol content was estimated as the sum of the particular fatty acid species of the assessed fraction and it was expressed in nanomoles per gram of the tissue.

### 2.9. Glycogen Concentration

Glycogen concentration in liver was measured by the method described by Kepler and Decker [29]. The liver samples were freeze-clamped in liquid nitrogen and further homogenized and then they were extracted with 8 mL of 6% HClO_4_. The supernatant was neutralized with 5 N K_2_CO_3_ and used for enzymatic glycogen assay [29].

### 2.10. Akt/PKB Expression

Akt/PKB expression was determined using Western Blot method. Routine Western blotting procedures were used to detect protein content as described previously [30]. The cells were lysed in ice-cold RIPA (radioimmunoprecipitation assay) buffer (50 mM Tris-HCl, 150 M NaCl, 1 mM EDTA, 1% NP-40, 0.25% Na-deoxycholate, 1 mM phenylmethylsulfonyl fluoride, 1 *μ*g/mL aprotinin, 1 *μ*g/mL leupeptin, 1 *μ*g/mL pepstatin, 1 mM sodium orthovanadate, and 1 mM sodium fluoride) and sonicated for 1 min at 4°C. Protein concentration was determined using BCA protein assay kit with bovine serum albumin as a standard. Samples were boiled at 95°C for 10 minutes in sample buffer containing 2-mercaptoethanol. Protein (60 *μ*g) was subjected to SDS-PAGE and transferred to PVDF membranes, followed by blocking membranes in TTBS buffer (50 mM Tris-HCl, 130 mM NaCl, and 0.05% Tween-20) containing 5% nonfat dry milk for 90 min at room temperature. The membranes were then incubated overnight at 4°C with the corresponding antibodies at a dilution of 1 : 1000. Primary antibodies were purchased from Cell Signaling Technology (Akt, phospho-Akt). Thereafter the membranes were incubated with anti-rabbit IgG horseradish peroxidase-conjugated secondary antibody (1 : 3000; Santa Cruz Biotechnology, USA). Immunoreactive protein bands were visualized by using an enhanced chemiluminescence substrate (Thermo Scientific, USA) and quantified by densitometry (Biorad, USA). Equal protein concentrations were loaded in each lane as confirmed by Ponceau staining of the blot membrane. Protein expression was normalized for *β*-tubulin and reported as arbitrary units. Finally, the control was set to 100% and the experimental groups were expressed relative to the control [30].

### 2.11. Statistical Analysis

Results are shown as mean ± standard deviation (SD). Statistical differences between groups (*n* = 8) were assessed using ANOVA with a subsequent post hoc test (Tukey HSD). Statistical significance was set at *P* < 0.01.

## 3. Results

### 3.1. Effect of Streptozotocin-Induced Diabetes and Myriocin Treatment on Body Weight, Plasma Glucose, Insulin, and FFAs Concentration (Table 1)

Average daily food intake was similar in all groups. Streptozotocin-induced diabetes caused about 30% (*P* < 0.01) reduction in body weight in D group compared with C group. Body weight decreased in both groups treated with myriocin (M and D + M) compared with C and D groups (*P* < 0.01), in about 20% and 40%, respectively.

Furthermore, streptozotocin-induced diabetes caused dramatic (*P* < 0.01) reduction in fasting plasma insulin concentration (below lower limit of detection) and concomitantly 4,3-fold (*P* < 0.01) elevation in fasting plasma glucose concentration in D group. Myriocin treatment did not affect fasting plasma glucose or insulin concentration in M group compared with C group. However, compared with D group, application of myriocin resulted in 50% reduction in fasting plasma glucose level in D + M group (from 504.6 ± 18.43 mg/dL to 254.6 ± 24.80 mg/dL).

The FFAs concentration increased by 66% and 78% (*P* < 0.01) in D and in D + M in comparison to C group. Myriocin treatment caused no changes in both M and D + M groups in relation to C and D groups, respectively.

### 3.2. Effect of Streptozotocin-Induced Diabetes and Myriocin Treatment on Sphingolipids Content in the Liver

**Figure 1:**
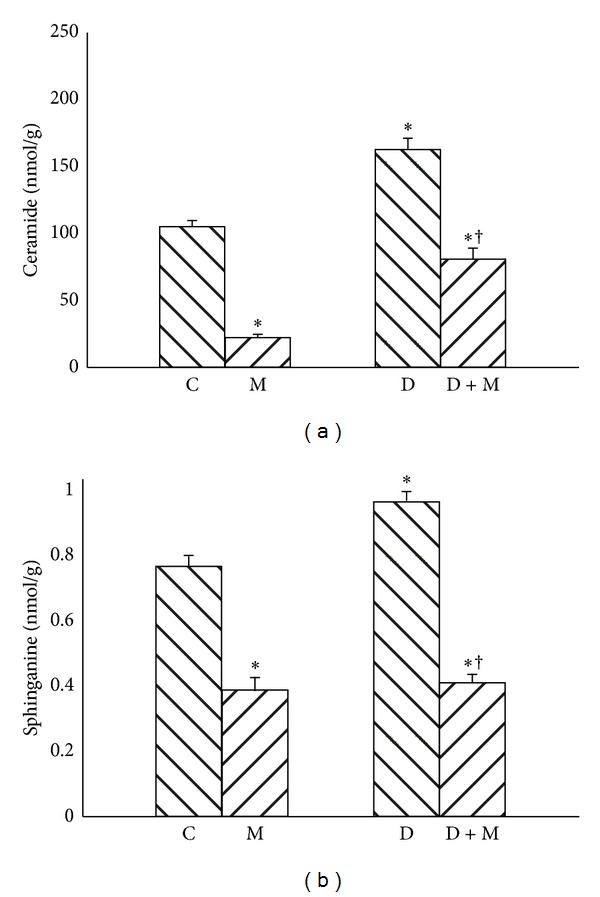
Effect of myriocin treatment (for 7 days) and streptozotocin-induced diabetes (for 7 days) on ceramide (a) and sphinganine (b) contents in liver homogenates. C: control group. M: group treated with myriocin. D: streptozotocin-induced diabetes group. D + M: streptozotocin-induced group treated with myriocin. Results are based on 8 independent preparations for each experimental treatment (means ± SE). *P < 0.01 compared with C group. ^†^ *P* < 0.01 compared with D group.

**Figure 2:**
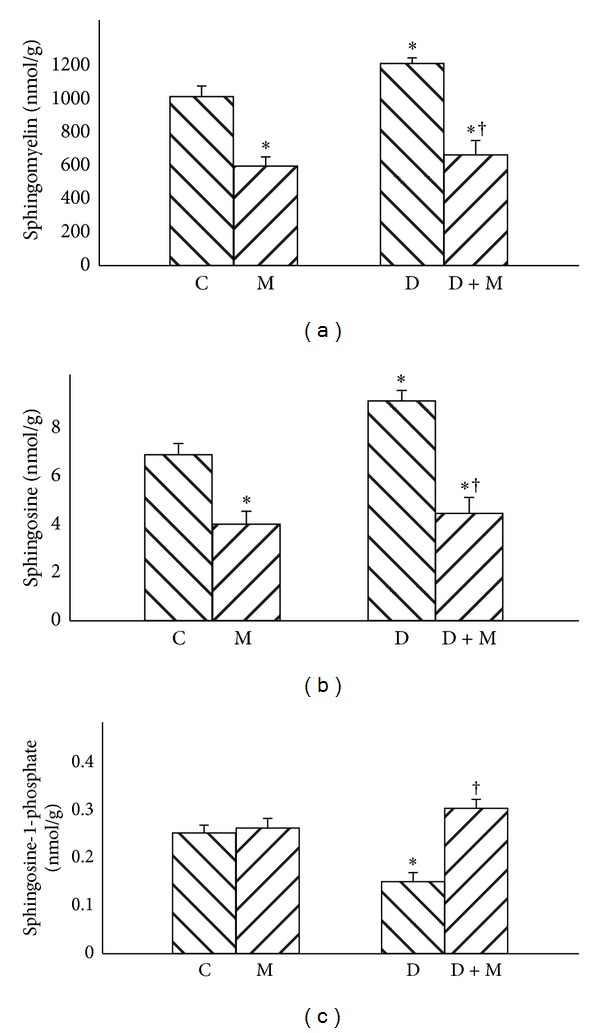
Effect of myriocin treatment (for 7 days) and streptozotocin-induced diabetes (for 7 days) on sphingomyelin (a), sphingosine (b), and sphingosine-1-phosphate (c) contents in liver homogenates. C: control group. M: group treated with myriocin. D: streptozotocin-induced diabetes group. D + M: treptozotocin-induced group treated with myriocin. Results are based on 8 independent preparations for each experimental treatment (means ± SE). *P < 0.01 compared with C group. ^†^ *P* < 0.01 compared with D group.

Compared with C group, rats in D group were characterized by 90% increase of ceramide and 28% increase of SFA content (*P* < 0.01) (Figures 1(a) and 1(b)). After myriocin treatment a reduction in ceramide level was noted (Figure 1(a), −75% and −65% resp., *P* < 0.01), accompanied by a significant decrease in sphinganine content (Figure 1(b), −50% and −60% resp., *P* < 0.01). Compared with C group, rats in D group were characterized by significant (+20%) elevation of sphingomyelin content (Figure 2(a), *P* < 0.01). Treatment with myriocin caused decrease in SM content in both M and D + M groups (Figure 2(a), −40% and −45%, resp., *P* < 0.01). Content of SFO was also increased in D group (+27%) and subsequently reduced after myriocin administration in both groups M and D + M (Figure 2(b), −37% and −53%, resp., *P* < 0.01). Compared with C group, S1P content was reduced −40% in D group (*P* < 0.01). On the other hand in D + M group level of S1P was significantly increased 1,13 fold (*P* < 0.01) in comparison to D group (Figure 2(c)).

### 3.3. Effect of Streptozotocin-Induced Diabetes and Myriocin Treatment on the Activity of Primary Enzymes Involved in Sphingolipid Metabolism

**Figure 3:**
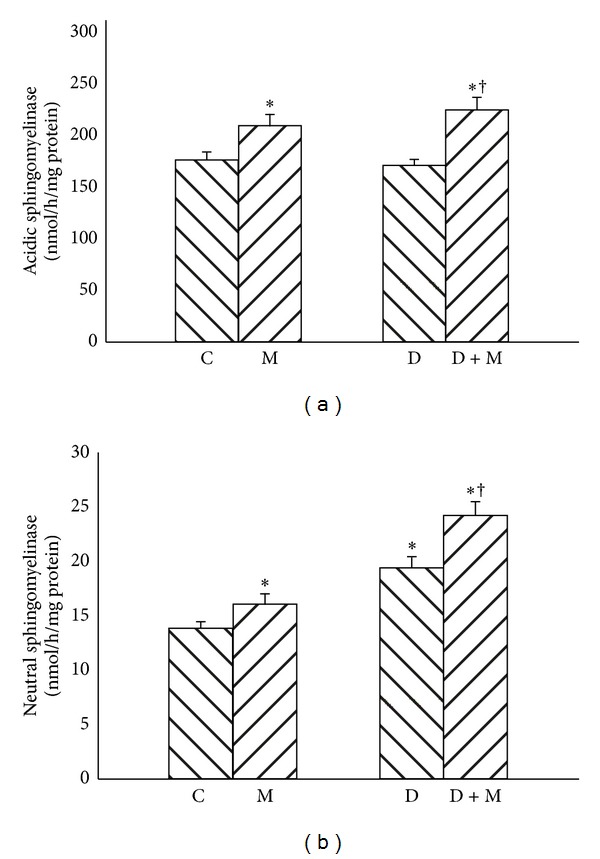
Effect of myriocin treatment (for 7 days) and streptozotocin-induced diabetes (for 7 days) on acidic sphingomyelinase (a) and neutral sphingomyelinase (b) activities in liver homogenates. C: control group. M: group treated with myriocin. D: streptozotocin-induced diabetes group. D + M: streptozotocin-induced group treated with myriocin. Results are based on 8 independent preparations for each experimental treatment (means ± SE). *P < 0.01 compared with C group. ^†^ *P* < 0.01 compared with D group.

Streptozotocin-induced diabetes in D group has only minor effect on the activity of acidic sphingomyelinase, but significantly increased activity of neutral sphingomyelinase compared with C group (Figure 3(b), +43%, *P* < 0.01). Furthermore, myriocin treatment increased the activity of acidic sphingomyelinase in groups M and D + M (Figure 3(a), +20% and +29%, resp., *P* < 0.01). The activity of neutral isoform of sphingomyelinase was also elevated in groups M and D + M (Figure 3(b), +18% and +21%, resp., *P* < 0.01).

**Figure 4:**
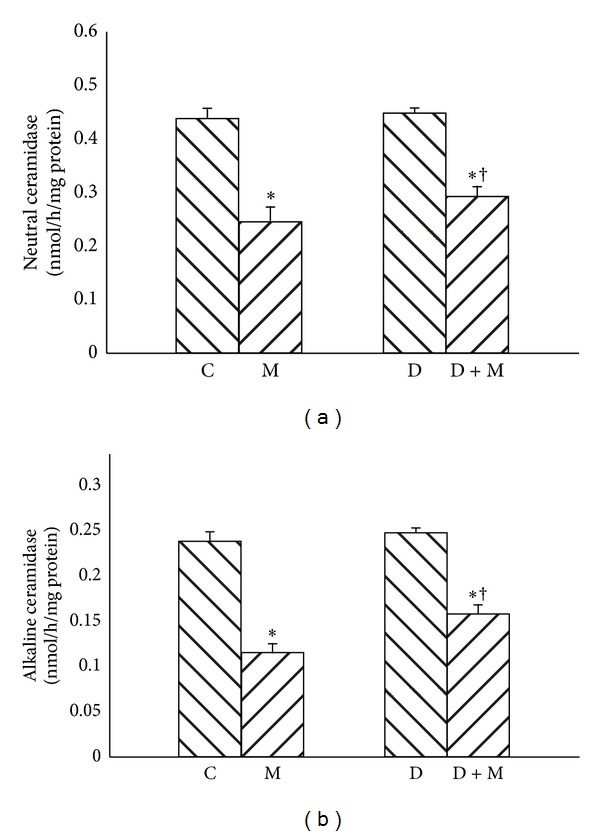
Effect of myriocin treatment (for 7 days) and streptozotocin-induced diabetes (for 7 days) on neutral ceramidase (a) and alkaline ceramidase (b) activities in liver homogenates. C: control group. M: group treated with myriocin. D: streptozotocin-induced diabetes group. D + M: streptozotocin-induced group treated with myriocin. Results are based on 8 independent preparations for each experimental treatment (means ± SE). *P < 0.01 compared with C group. ^†^ *P* < 0.01 compared with D group.

No changes in neutral or alkaline ceramidase activities between streptozotocin-induced diabetes group and control group were observed. However, myriocin treatment resulted in decreased activity of neutral (Figure 4(a), −42% and −33%, resp., *P* < 0.01) and alkaline ceramidase (Figure 4(b), −50% and −31%, resp., *P* < 0.01) in groups M and D + M.

### 3.4. Effect of Streptozotocin-Induced Diabetes and Myriocin Treatment on DAG and TG Content in the Liver

**Figure 5:**
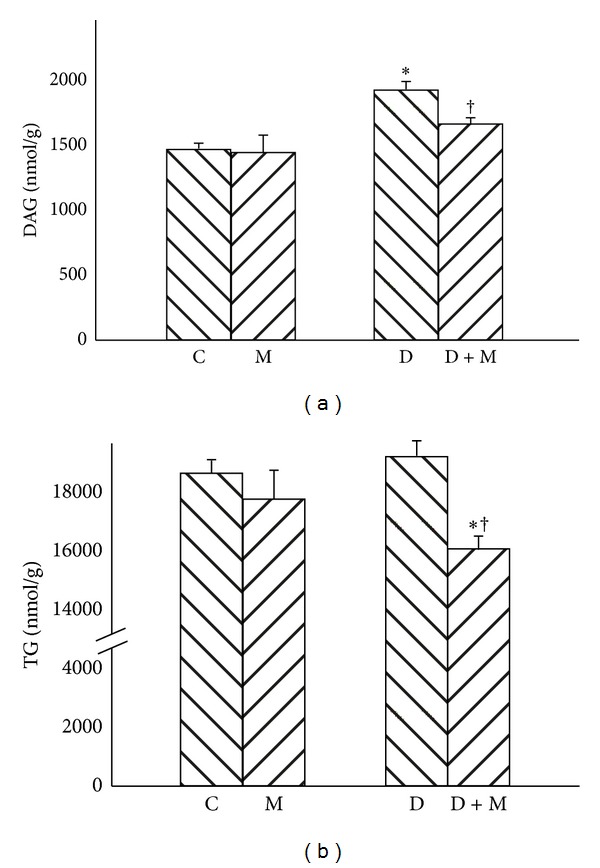
Effect of myriocin treatment (for 7 days) and streptozotocin-induced diabetes (for 7 days) on DAG (a) and TG (b) contents in liver homogenates. C: control group. M: group treated with myriocin. D: streptozotocin-induced diabetes group. D + M: streptozotocin-induced group treated with myriocin. Results are based on 8 independent preparations for each experimental treatment (means ± SE). *P < 0.01 compared with C group. ^†^ *P* < 0.01 compared with D group.

Streptozotocin-induced diabetes resulted in 32% elevation of DAG content in comparison with C group (Figure 5(a), *P* < 0.01). Administration of myriocin caused significant reduction of DAG quantity in D + M group, almost to the level observed in C group (Figure 5(a), *P* < 0.01). There was no significant difference in the liver TG content between C, M, and D groups (Figure 5(b)); however, myriocin treated rats from D + M group were characterized by reduced TG concentration in comparison with C and D groups (Figure 5(b), −15% and −19%, resp., *P* < 0.01).

### 3.5. Effect of Streptozotocin-Induced Diabetes and Myriocin Treatment on Glycogen Concentration and Akt/PKB Expression in the Liver

**Figure 6:**
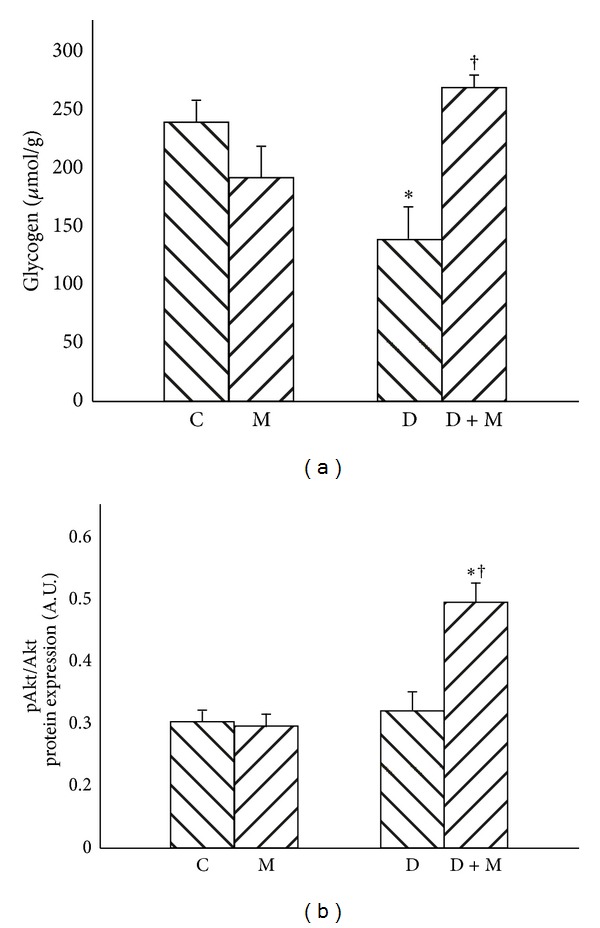
Effect of myriocin treatment (for 7 days) and streptozotocin-induced diabetes (for 7 days) on glycogen content (a) and Akt/PKB expression (b) in liver homogenates. C: ontrol group. M: group treated with myriocin. D: streptozotocin-induced diabetes group. D + M: streptozotocin-induced group treated with myriocin. Results are based on 8 independent preparations for each experimental treatment (means ± SE). *P < 0.01 compared with C group. ^†^ *P* < 0.01 compared with D group.

Streptozotocin-induced diabetes markedly decreased liver glycogen level in comparison with C group (Figure 6(a), *P* < 0.01). However, application of myriocin resulted in significant elevation of glycogen concentration in D + M group (Figure 6(a), *P* < 0.01).

There were no significant changes of Akt/PKB expression between all groups. Furthermore, there were no differences in phospho-Akt/PKB (pAkt/PKB) expression between C, M, and D groups; however, administration of myriocin resulted in significant elevation of pAkt/PKB in D + M group (Figure 6(b), *P* < 0.01).

## 4. Discussion

Our experiment was carried out on male Wistar rats with diabetes induced by streptozotocin, which, since the early 1970s, is one of the most commonly used experimental models of type 1 diabetes [31]. In our study, in line with expectations, we observed severe hyperglycemia and concomitantly very low fasting plasma insulin level (below the lower limit of detection) after streptozotocin injection [32]. This confirms that within a week, after administration of streptozotocin, pancreatic *β*-cells were destroyed and consequently endocrine insufficiency of the pancreas was developed. Furthermore, diabetic animals were characterized by significantly decreased body mass. Moreover, after myriocin treatment we noticed subsequent weight loss, which was observed in spite of similar food intake in all groups of the animals. In the other study performed on diet induced rodent model of diabetes, Yang et al. [33] suggested that body weight reduction after myriocin treatment depends on improved leptin signaling [33]. It seems like a plausible explanation since leptin regulates central and peripheral signaling pathways responsible for energy consumption. On the other hand, Ussher et al. [34] did not observe any weight loss due to myriocin treatment; perhaps this disagreement may result from the different time of myriocin administration in both cited studies.

Data published so far have proven that one of the most important pathogenic factors in diabetes onset is a dysregulation of hepatic lipid metabolism, which leads to elevation of hepatic ceramide content [35]. In accordance with this statement, the present study noted higher hepatic concentrations of ceramide and other sphingolipids (such as SM and SFO), which is also consistent with other published data [31]. In our survey intraperitoneal administration of SPT inhibitor—myriocin—caused several significant changes in sphingomyelin signaling pathway. First of all, we observed, that myriocin treatment significantly reduced ceramide *de novo* synthesis in the liver. This was manifested primarily by dramatic reduction of ceramide level and by decrease of SFA (an important substrate for ceramide *de novo* synthesis) content. Additionally, myriocin treatment inhibited activity of neutral and alkaline ceramidases. It seems therefore that this, observed after myriocin administration, reduction of ceramide content is *per se* a result of inhibition of its *de novo* production and does not depend on changes in ceramidases activities. Furthermore, we have noticed the reduction of SFO (ceramide downstream metabolite) content, which is consistent with a well-established role of myriocin in ceramide *de novo* synthesis and which reflects a reduction of ceramidases activities and decreased ceramide content. Interestingly, we have observed significant reduction in S1P content in case of streptozotocin-induced diabetes, which is even more interesting given the fact that S1P stimulates pancreatic *β*-cells growth and promotes insulin secretion. Furthermore, after myriocin treatment, S1P hepatic level was significantly increased. This observation confirms a beneficial role of ceramide metabolite S1P in glucose homeostasis. In contrast to our study, Yang et al. [33] demonstrated modestly decreased S1P plasma concentration in myriocin treated mice [33]. However, in this survey longer period of SPT inhibitor administration was applied, which could explain the observed differences. Moreover, we found that myriocin treatment has also decreased hepatic SM level. Since myriocin stimulates activity of both acidic and neutral sphingomyelinase isoforms, decrease in SM content in the groups treated with myriocin may be a result of this activation.

One of the most important findings in our study is that inhibition of ceramide *de novo* synthesis reduces glucose plasma concentration in type 1 diabetic rats. Several different mechanisms could potentially explain this phenomenon. First of all ceramide inhibits glycogen synthesis in hepatocytes [9]. This feature has been observed in previous studies, where rats with short-term and long-term type 1 diabetes were characterized with markedly reduced liver glycogen content [36, 37]. In the present paper we have demonstrated similar findings. In order to determine whether, observed in course of diabetes, reduction of liver glycogen concentration is related to ceramide content we decided to measure the level of glycogen in hepatocytes also after myriocin administration. Interestingly, we found that reduction of ceramide level by inhibition of its *de novo* synthesis has led to enhanced hepatic glucose accumulation in the form of glycogen. This further translates to decreased glucose plasma level in peripheral blood. Contrarily to our results, studies performed by van Sluijters et al. [38] revealed that sphingomyelinase inhibits glycogen synthesis by inhibition of glycogen synthase activity in rat hepatocytes [38]. However, the above cited authors did not measure ceramide content in hepatocytes.

Another important mechanism, by means of which inhibition of ceramide *de novo* synthesis ameliorates glucose homeostasis, is the liver insulin resistance. It has been previously demonstrated that streptozotocin treated rats show increased ceramide content in skeletal muscle samples [39]. These changes were accompanied by increased SPT and neutral SMase activity, which indirectly indicates increased rate of ceramide synthesis. As mentioned above ceramide antagonizes insulin signaling *via* suppression of Akt/PKB phosphorylation and inhibition of GLUT-4 translocation in the liver. Moreover, ceramide directly activates protein phosphatase 2A, an enzyme responsible for deactivation (dephosphorylation) of Akt/PKB [40]. These aforementioned mechanisms finally lead to impairment of insulin action in hepatocytes. To determine whether in our experiment ceramide accumulation in hepatocytes led to development of the liver insulin resistance we measured Akt/PKB expression. However, we observed only a trend which did not reach statistical significance in Akt/PKB expression in myriocin treated group, but surprisingly we found that administration of myriocin resulted in significant increase of pAkt/PKB expression. Thus, it seems that inhibition of ceramide *de novo* synthesis increases Akt/PKB phosphorylation, thereby enhancing GLUT-4 translocation and glucose uptake in hepatocytes. Subsequently, myriocin treatment reversed hepatic insulin resistance and lowered fasting plasma glucose level.

Another important factor responsible for diabetes onset and progression is free fatty acids. Plasma level of FFAs in the group of rats with streptozotocin-induced diabetes was significantly higher in relation to the control group. The increase of FFAs in diabetic animals' blood plasma is in accordance with previous literature data [15, 39]. Free fatty acids regulate mechanisms conditioning impaired glucose uptake and glycogen synthesis, which in turn lead directly to hyperglycemia observed in the presented group. However, no studies evaluating the effect of myriocin on the level of FFA were performed so far, so it is impossible to relate our results to the literature. We observed in our study a slight increase in free fatty acids content that may be due to the inhibition of ceramide *de novo* synthesis pathway. FFAs, not used in the synthesis of ceramide, could therefore accumulate in different cells (e.g., hepatocytes) and tissues.

Furthermore, another interesting and novel finding in our study is that application of myriocin resulted in a reduction of hepatic liver DAG and TG levels. As expected, animals with streptozotocin-induced diabetes were characterized by significant elevation of DAG content in the liver. Interestingly, inhibition of ceramide *de novo* synthesis resulted in decreased DAG level. Moreover, this reduction was also associated with amelioration of hepatic glucose homeostasis. Moreover, biochemical analysis revealed reduced hepatic TG level in group of streptozotocin-induced diabetic rats treated with myriocin. These are advantageous findings because TG stored in the liver in a form of lipid droplets leads to hepatocyte necrosis and induces its inflammation and their serious consequences. Previously published studies appear to strongly indicate that intrahepatocyte accumulation of toxic lipids (i.e., DAG and TG) negatively affects insulin signaling pathway. We presented in our survey observations that are consistent with results achieved by the other authors in their studies of animal models of type 2 diabetes [24, 33]. Moreover, Dekker et al. [41] have recently proved that inhibition of sphingolipid synthesis with myriocin improves dyslipidemia in the diet-induced hamster model of insulin resistance [41].

In summary, although it is well established that inhibition of ceramide *de novo* synthesis reverses obesity-induced insulin resistance [24, 33, 41], presence of this phenomenon in case of type 1 diabetes was not sufficiently explored so far. In our study we have showed, for the first time, that inhibition of ceramide *de novo* synthesis with SPT inhibitor—myriocin—ameliorates glucose homeostasis in streptozotocin-induced type 1 diabetes. Another novel finding of our research is that myriocin application increased S1P concentration in the liver. Thus, it can be considered as a potential new therapeutic tool for type 1 diabetes treatment in the future.

## Figures and Tables

**Table 1:** Effect of streptozotocin-induced diabetes and myriocin treatment (for 7 days) on body weight, fasting serum glucose, insulin, and FFA levels.

	C	M	D	D + M
Body weight (g)	305,8 ± 18,00	246,0 ± 16,93*	215,2 ± 10,85*	188,2 ± 12,09^∗†^
Glucose level (mg/dL)	102,8 ± 5,93	92,8 ± 6,34	536,0 ± 16,26*	254,6 ± 24,80^∗†^
Insulin level (µU/mL)	4,5 ± 0,81	4,8 ± 0,64	nd	nd
FFA level (µmol/L)	88,6 ± 10,48	95,8 ± 8,89	150,4 ± 17,30*	156,8 ± 12,4*

C: control group; M: group treated with myriocin; D: streptozotocin-induced diabetic group; D + M: streptozotocin-induced diabetic group treated with myriocin; FFA: free fatty acids. Results are based on 8 independent preparations for each experimental treatment (means ± SE).

*P < 0,01 compared with C group. ^†^ *P* < 0,01 compared with D group. nd: not detected.
